# Deconstructing noncompliance: parental experiences of children’s challenging behaviours in a clinical sample

**DOI:** 10.1080/17482631.2018.1563432

**Published:** 2019-01-11

**Authors:** Jane Robson, Leon Kuczynski

**Affiliations:** Department of Family Relations and Applied Nutrition, University of Guelph, Guelph, ON, Canada

**Keywords:** Child agency, noncompliance, resistance, aggression, socialization, social relational theory (SRT)

## Abstract

**Purpose:** This study explored the phenomenon of children’s nonconforming behaviours from the perspective of parents who sought clinical services for children’s severe noncompliance. **Method:** Mothers from 25 families who accessed clinical services were interviewed about their relationship with their children aged 8–13 and their experiences of their children’s challenging behaviours. **Results:** Mothers distinguished two different types of challenging behaviour: normative resistance and extreme aggression. Mothers described normative resistance as an expected part of children’s developing autonomy and treated resistance with behavioural management strategies. Mothers also described occasions when children displayed emotionally dis-regulated extreme aggression, which were consistent with clinical descriptions of children’s difficult to manage behaviour. **Conclusion:** Contrary to clinical recommendations mothers used relational strategies to reconnect children with their agency. The distinction between two different child behaviours, and strategies for each challenging behaviours have theoretical and practical implications.

Children’s propensity to express their agency by resisting or disobeying the requests and rules of their parents is a common phenomenon of family life, a central focus in research on socialization and a frequent target of interventions in the family (Kalb & Loeber, 2003; Patterson, 1982). It is difficult to identify another childhood phenomenon that has been subjected to a more diverse array of social and theoretical constructions. Underlying these conceptions are different social discourses about the value and legitimacy of children’s agency and influence in the family emanating from culture, social class, and religion (Kuczynski, Lollis, & Koguchi, 2003; Trommsdorff & Kornadt, 2003). In their review of the socialization literature, Kuczynski and Hildebrant (1997) identified multiple conceptions of children’s nonconformity including: *willful defiance* (authoritarian perspective), *noncompliance* (behavioural perspective), *cognitive non-acceptance* (internalization perspective) *unresponsiveness (*attachment perspective), and *resistance* (developmental perspective). Each of these theoretical constructs offer different ideas about the aetiology of children’s resistance, different views about the role children’s resistance in children’s health and well-being, and different directions for parents regarding how to interpret and handle children’s nonconforming behaviour.

In this article, we explore children’s nonconforming behaviours from the perspectives of mothers who have accessed clinical services for support in handling the difficult to manage and aggressive behaviours of their children. Children’s noncompliance is the most frequent presenting problem for parents who seek the help of clinical and mental health services. In this literature noncompliance is associated with a complex array of child and family environment factors. Noncompliance is an indicator for diagnoses such as conduct disorder (Dadds, Sanders, Morrison & Rebgetz, 1992), oppositional defiant disorder (Burke, Loeber, & Birmaher, 2002) and attention deficit hyperactivity disorder (Cantwell, 1996). Family factors associated with severe noncompliance include high stress, poverty, low education and the presence of substance abuse. (Matthys & Lochman, 2017; Patterson Reid & Dishion & 1992).

The behavioural perspective, on children’s nonconformity (Patterson, 1982) has dominated the applied literature on clinical interventions in the family. In the behavioural perspective children’s nonconformity is conceptualized as *noncompliance* which is defined as the failure to comply exactly and immediately with the parents’ request. Coercive process theory (Patterson, 1982; Patterson et al., 1992) provides the conceptual background for targeting noncompliance as a focus of interventions in the family. In this theory noncompliance is an aversive behaviour that plays a key role in initiating and maintaining reciprocal escalating coercive cycles of interaction between parents and children. The assumption is that if the parent does not suppress noncompliance, the parent and the child will then be more likely to exchange aversive behaviours at a higher intensity until the parent or child withdraws and terminates the aversive interaction. Although children are considered to be agents in this theory, child agency is conceptualized in the limited and unconstructive sense of evading compliance and contributing to reactive mutually noxious patterns of behaviour (Hollenstein, Granic, Stoolmiller, & Snyder, 2004; Patterson et al., 1992).

Developmental research provides a deeply contrasting view that emerged from the study of children’s nonconforming behaviour as a frequently occurring phenomenon in non-clinic and, presumably, well-functioning families. In this view children’s nonconforming behaviour is conceptualized as *resistance* a healthy expression of children’s agency in the parent–child relationship. The origins of this view can be traced to research on the phenomenon of toddler negativism in the 1930’s (Wenar, 1982). In these accounts, the emergence of “no” was conceptualized as a healthy manifestation of children’s developing autonomy as young children pushed back on their parents increasing attempts to control their actions. Further work on this concept was undertaken by researchers who explored the development of children’s resistance strategies using nonclinical samples. In contrast, to the behavioural view of noncompliance as a negative behaviour that parents should suppress, in developmental research, children’s resistance is viewed more positively as an opportunity for parents to support their children’s autonomy when appropriate (Kochanska & Kuczynski, 1991) and guiding their children to develop social skills for expressing their autonomy in an socially competent manner.

Currently, children’s noncompliance strategies in North American samples has been studied covering the periods of early childhood (Crockenberg & Litman, 1990; Kuczynski & Kochanska, 1990; Kuczynski et aI., 1987) middle childhood (Kuczynski, Pitman & Twigger, (this volume) and adolescence (Parkin & Kuczynski, 2012). In this research children’s resistant behaviours are interpreted as interpersonal influence strategies that vary along dimensions of assertiveness, social skill and overt versus covert mode of expression.

These variations in expressing resistance are important because parents have been found to respond to children’s resistance in a highly contextualized manner that depends on the nature of the social situation (Grusec & Davidov, 2010; Smetana, 2006). Moreover, there is a growing body of research indicating that found that only oppositional forms of noncompliance are associated with negative outcomes for children (Crockenberg & Litman, 1990; Drabick, Strassberg, & Kees, 2001; Kuczynski & Kochanska, 1990) and that assertive forms of resistance may be associated with positive parenting practices (Dix, Stewart, Gershoff, & Day, 2007; Morrissey & Gondoli, 2012)

In Canada, parents who seek help for difficult to manage noncompliant behaviours are often referred to family support systems, such as parenting training programs, in an attempt to reduce children’s noncompliant behaviours (Barkley, 1987; Burke et al., 2002; Dishion et al., 2008). Specific programs, such as the Triple P: Positive Parenting Program (Sanders, 1999), or the Incredible Years Parenting Program (Webster-Stratton, 2011), are informed by behavioural conceptions of the presenting problem. Consequently, the focus of behavioural parent-training programs is on teaching parents how to authoritatively communicate commands and enforce them with negative consequences including time outs if children do not respond to the command (McMahon & Forehand, 2003). A common recommendation in these programs is that parents should be trained to define children’s noncompliance as a coercive behaviour and suppress noncompliant children’s behaviours immediately after they occur (Forgatch, Beldavs, Patterson, & DeGarmo, 2008). Despite evidence of success for interventions that use the behavioural definition of noncompliance (Graaf, Speetjens, Smit Wolff, & Tavecchio, 2008) it remains the case that alternative conceptualizations of children’s nonconformity have been mostly ignored in the clinical literature.

Parents seeking help for problematic levels of noncompliance are likely to be exposed to one of two contradictory theoretical frameworks for understanding and treating the behaviour. These are clinical frameworks which conceptualize nonconforming behaviour using the problem focused lens of *noncompliance*, a deviant behaviour that needs to be suppressed and developmental frameworks that conceptualize children’s nonconforming behaviour as *resistance* a legitimate act of agency that requires guidance. The lack of integration of two contradictory evidence-based perspectives on children’s noncompliance or resistance to parental requests is problematic for both theory, parental education and clinical interventions. These two bodies of literature currently inform two very different approaches to parenting that are currently considered to be mutually exclusive. One source of this difference may be at the meta-theoretical level which fundamentally guides research. Behavioural perspectives are guided by a mechanistic or linear model of socialization that perceives children’s behaviours to be passive or reactive outcomes of parental forces whereas developmental perspectives are guided by an organismic perspective that perceives children as inherently active, self-organizing, meaning making agents (Kuczynski & De Mol, 2015). Another reason for lack of integration may be empirical and has to do with the nature of the populations from which the different perspectives on agency have emerged. The problem focused concept of noncompliance emerged from research on clinical populations where parents seek help for managing children’s uncooperative behaviours. In contrast the developmental concept of resistance has thus far been completely based on non-clinical families where the assumption is that children’s uncooperative behaviours lies within a normal and manageable range. This means that noncompliance and resistance may be distinct constructs theorized for qualitatively different phenomena. Consistent with this argument, major clinical reviews on noncompliance (Matthys & Lochman, 2017; McMahon & Forehand, 2003) have acknowledged developmental research but largely dismiss their relevance for clinical populations.

The purpose of this study is to gain a better understanding of the phenomenon of children exhibiting noncompliant behaviours from the perspective of parents who seek clinical services for these behaviours. There is little literature that explores clinical parents’ lived experiences of the presenting problem of difficult to manage challenging behaviours and existing research to date has been conducted solely using a problem focused-behavioural lens. The research strategy was to explore the phenomenon of child nonconformity in a clinical sample using sensitizing ideas deriving from both behavioural and developmental perspectives.

## Method

### Recruitment

Ethics approval was received from the Research Ethics Board at the University of Guelph (15AP009). The participants of this study were mothers of children between the ages of eight to 13 who had accessed parenting support systems for support concerning their children’s challenging behaviours. Parenting support systems were defined broadly as agencies or organizations that provided formal training or support programming for parenting children with noncompliant or resistant behaviours. A local non-for profit counselling agency helped in recruitment by circulating recruitment posters to parents accessing one-on-one and group support for parenting children exhibiting noncompliant behaviour. Compensation was provided to participants in the form of a $10 gift card to a location of their choice.

### Participants

Twenty-five mothers participated in this study. Mothers were asked to complete a demographic survey before the interview. The mothers in this study lived in southern Ontario. Their average age was 39 and ranged from 28 to 50 years. There were 16 mothers who identified as White/European, three who identified as Black/African/Caribbean, two who identified as Arab, two who identified as South Asian, one who identified as Canadian/Irish and one who identified as Latin American. There were 15 mothers who had graduated from college or university, six mothers who had completed high school and four mothers that had completed graduate education. Twelve of the mothers stated they were single, nine of the mothers were married, and four of the mothers were living with a common law partner.

The ages of the target children ranged from eight to 13, with an average age of 10 years. Thirteen of the children were male and twelve of the children were female. Nineteen of the children had siblings and six of the children did not. Each of these mothers stated that they had accessed support services for the presenting problem of challenging child behaviours. The services accessed by mothers varied and included family therapy, individual therapy, group therapy, or psycho-educational classes reflecting attachment and behavioural orientations.

Eighteen children had been formally diagnosed (American Psychiatric Association, 2013), with classifications that included: Asperger Syndrome, Attention Deficit Disorder, Attention Deficit Hyperactivity Disorder, Anxiety, Conduct Disorder, Depression, Gifted, Oppositional Defiant Disorder, Sensory Process Disorder, Sleep Disorder, and Unspecified Learning Disability. The range of diagnoses reflects the diversity of conditions in which noncompliance can be a presenting problem.

### Interview

Mothers participated in an interview that took one to two hours. The interview had three parts, the nature of their relationships with their children, parent–child interactions pertaining to children’s noncompliance and mothers’ experiences of the clinical services that they received. For the purposes of the present study only the data on noncompliance are presented. Mothers were asked to describe two kinds of circumstances: typical events during the past week when the child did not comply with her directions and less-frequent incidents that were challenging or harder to manage.

### Data analysis

Interviews were transcribed and analyzed using constructivist grounded theory methodology (Charmaz, 2003). Constructivist grounded theory (CGT) emphasizes the influence of the researcher, participants and their relationship on the analysis and interpretation of data (Charmaz, 2006). In CGT Charmaz (2006) argues that the process of gathering and interpreting data is never neutral, rather researchers can position themselves as participants in the construction of understanding. This enables researchers to be focused on interpreting the meanings of process or experience, rather than attempting to access a single truth (Charmaz, 2003). A CGT approach encourages researchers to reflect on the influence of their knowledge, interests and theoretical orientations in conjunction with their relationship with research participants (Daly, 2007). Participants are considered active partners in the research process, with the capacity to negotiate, process and reflect on their lived experiences (Charmaz, 2006).

The initial stages of the analyses were sensitized by ideas from behavioural theories and developmental theories of socialization processes. However deliberate care was taken to ensure that the codes reflected mothers’ narratives especially when they departed from existing ideas in the literature. Throughout the process of initial, open, and focused coding, the first author used memos to elaborate actions, assumptions and processes which are subsumed in data and code (Charmaz, 2006). The codes that made the most analytic sense were elevated to categories, and enabled us to organize data in a more complete and accurate manner.

Analyses were aided by qualitative data analysis software program, MAXQDA, in order to ensure the systematic categorization of data and documentation of the analytic process in memos. Also, to ensure the trustworthiness of the analyses first and second author met regularly to review the emerging themes, discuss alternative interpretations, and to ensure rigour in the constant comparison process.

## Results

All mothers in this clinical sample reported that their children displayed two qualitatively different kinds of challenging behaviours at different times: *normative resistance* and *extreme aggression*. As can be seen by comparing Tables I and II, the nature of the child behaviours, mothers’ explanations of the behaviours, and mothers’ strategies for managing the behaviours differed markedly for these two categories. Normative resistance referred to nonconforming actions that mothers interpreted as a developmentally expected expression of children’s autonomy that was intentional and well-regulated; extreme aggression referred to nonconforming behaviours that mothers interpreted as reactive, out of control and emotionally dis-regulated.

**Table I.** Mother’s perceptions, attributions and responses to normative resistance.

Categories	Number of mothers (N = 25)
Normative resistance	
Resistance	24
Displaying attitude	20
Parental attributions	
Personality	11
Normalizing resistance	17
Parental responses	
Being proactive	12
Firm enforcement	17
Promoting skilful autonomy	15

**Table II.** Mother’s perceptions, attributions and responses to extreme aggression.

Categories	Number of mothers (N = 25)
Extreme aggression	
Destroying property	17
Physical aggression	19
Verbal coercion	20
Self-harm	6
Parental attributions	
Mental health	10
Losing control	18
Parental responses	
Verbally reassuring	8
Physically reassuring	10
Making relational contact	8

### Normative resistance

Mothers reported that normative resistance to their requests and rules was a part of their daily experiences and interactions with their children. Mothers described routine acts of resistance such as refusing to follow parental requests that mothers accepted as a normal part of the mother–child relationship. The two sub-categories evident related to normative resistance were *overt resistance* and *displaying attitude.*

### Overt resistance

Mothers described four strategies used by their children to overtly express resistance to their requests: refusing, ignoring, negotiating, and delaying. Mothers reported that these forms of resistance were expected occurrences that occurred regularly. For example, one mother said, “Refusing something, or, it’s like ‘I’m not going to school tomorrow, I’m in a really bad mood’ or ‘I hate it. You know I have no friends. I’m not going’” (Family 15, 11-year-old daughter). Another mother reported “I ask him to move them and he won’t…I’ve asked him three times, he didn’t do it” (Family 2, 8-year-old son).

Mothers also reported that rather than assertively challenging them, their children resisted indirectly without acknowledging their requests. For example, one mother reported when her daughter was asked to complete a chore, “She just ignores” (Family 12, 13-year-old daughter). Another mother described an incident of her son ignoring her when asked to take out the garbage, “There’s definitely some times where I feel like I am purposely being ignored, … and it’s just like [sighs]” (Family 21, 10-year-old son).

Mothers also frequently reported that their used negotiation to evade complying or to suggest compromises regarding the timing or amount of compliance. For example, one mother stated “Well, she will ask me things like, ‘if I finish this up early, does that mean I can go to my friend’s early?’ and I think that’s fair” (Family 10, 12-year-old daughter). Other mothers reported children using explanations as a delaying tactic. For example, rather than complying immediately with a request to getting off his gaming station, one mother reported, “So he is like, ‘just one more minute, just one more second, I just have to pass this one level’ (laughs), it’s always something. Isn’t there always one more level” (Family 25, 11-year-old son).

### Displaying attitude

Most mothers reported that their children complied with a request but challenged parental authority with verbal or nonverbal expressions of non-acceptance. Attitude was described by mothers as the child being “surly and sassy” (Family 11, 13-year-old son) or “snarky” (Family 2, 8-year-old son). Mothers reported that children expressed attitude by “talking back” (Family 17, 12-year-old son), “rolling their eyes” (Family 16, 12-year-old daughter), or “being rude” (Family 20, 10-year-old daughter).

Mothers also reported being subjected to displays of attitude when they enforced their demands and compelled their children to comply. For example, one mother explained her daughter displaying attitude when she was trying to get her to put her put a dish away, “So she puts it away, like not nicely though… she will sigh really loud… or that whole ‘Mom!’ whine” (Family 16, 12-year-old daughter). Another mother reported, “She gives me that knowing look like, “Yeah, you are right, I know we have already talked about this. I don’t like it, but I get it. Mom, I’ll do it”’ (Family 20, 10-year-old daughter).

### Parental explanations for normative resistance

Mothers contextualized their reports of normative resistance by spontaneously offering explanations that attributed the behaviour to normal developmental processes. These explanations took two forms, *personality attributions* and *normalizing resistance*.

#### Personality attributions

Many mothers explained that their child’s expression of normative resistance was due to inherent characteristics that were a part of the child’s personality or temperament. For example, one mother reported, “He’s got a very zesty character and I didn’t want to take that away from him. I just wanted him to be able to function and for me to function” (Family 13, 13-year-old son). Another mother reported that her son’s resistance was due to his “oppositional character… since he was small” (Family 3, 12-year-old son). These examples suggested that mothers perceived resistant behaviour to be manifestations of normal variations in children’s unique temperament

#### Normalizing resistance

Mothers also attributed incidents of normative resistance to developmental norms and milestones. Several examples of these attributions to normal processes are provided. “She’s at that age so some of this is probably pretty normal” (Family 10, 12-year-old daughter). “It’s gotten more challenging as he’s hitting puberty, but with all the change, and emotions and the anger coming up, um, it’s been a lot more challenging with him” (Family 23, 11-year-old son). “I guess it’s her age or the age of them, attitude and peer pressure” (Family 12, 13-year-old daughter). “She is obviously a teenager now, and so it’s like the terrible twos time but it’s more for like, teenagers, right?” (Family 16, 12-year-old daughter). These narratives suggest that although mothers regarded resistance to be irritating or annoying such forms of nonconformity and opposition were expected signs of their children’s growing autonomy.

### Parental responses to normative resistance

Mothers reported that they responded to their children’s everyday resistance using a range of strategies for managing children’s behaviour. These strategies included *being proactive, firm enforcement*, and *promoting autonomy.*

#### Being proactive

Mothers reported that they used proactive problem solving strategies to ward off children’s normative resistance to their requests. One approach was to put in place rules in the attempt to anticipate problems and prevent uncooperative behaviour. For example, many mothers reported issues with their children spending too much time on technology such as tablets, phones and gaming systems. One mother reported that when her child began to resist getting off their tablet, she would plan with the child a clear schedule for when and how long they could use technology in the future. This mother reported, “I guess you could say we are trying to be preventative by setting up rules” (Family 22, 10-year-old son).

Mothers also reported that they proactively created routines or schedules to minimize resistance in future interactions. One mother provided an example of a routine she developed in response to her child delaying tasks and chores. She stated, “We come up with a weekly schedule for him so that he knows what is happening regularly and anything unusual like a doctor’s appointment or something is written on his schedule” (Family 9, 9-year-old son).

#### Firm enforcement

Mothers reported that when their child resisted a rule or did not fulfil an expectation, they firmly enforced their expectations. Mothers said they “make it clear” (Family 16, 12-year-old daughter) to the child what was expected, and “be really direct” (Family 3, 12-year-old son) about how the child could successfully fulfil that expectation. For example, one mother reported,

I have to push him to do and it’s usually a battle every single time. It’s funny because he does the same things ever single Saturday. He has a list that is step by step, but every single time I have to inspect and be like you forgot to do this, did you do this. (Family 2, 8-year-old son)

Mothers emphasized the importance of firm consistent firm enforcement for reducing resistance in the long term. For example, one mother said she would tell her son, “Sit down and eat your fruit, sit down and eat your cereal, sit down, sit down, sit down. The consistency and following through is so important during that time” (Family 3, 12-year-old son). Another mother explained, “I think making her repeat it and talk about it is hopefully planting that seed that will grow eventually” (Family 5, 13-year-old daughter).

#### Promoting autonomy

Mothers interpreted children’s typical expressions of resistance as manifestations of their children’s growing autonomy. Some mothers said that when the issues are not crucial they supported their children’s autonomy by tolerating resistance or encouraging their children to express resistance in a skilful or socially appropriate manner. For example, one mother reported that she encouraged her daughter to be assertive through resistance. The mother said that her daughter “is learning how to stand up for herself and how to ask for what she wants… That is something that we have been working on, and that, I think, is important for kids” (Family 20, 10-year-old daughter). However, the same mother focused on guiding her child’s expression of agency by encouraging her daughter to express resistance in a skilful way: “don’t be rude, don’t be disrespectful” (Family 20, 10-year-old daughter). This mother stated that if resistance was expressed skilfully and respectfully, she was open to adjusting her expectations for her daughter.

### Extreme aggression

Mothers reports regarding the most challenging or difficult to manage nonconforming behaviours were classified as extreme aggression. These instances occurred less frequently, were perceived as having different causes and were handled in a qualitatively different manner than normative resistance. Mothers described four categories of extreme aggression—*destroying property, physical aggression, verbal coercion*, and *self-harm*. Mothers described extreme aggression using language such as “defiant,”, “oppositional”, “aggressive”, “explosive”, “destructive” and “violent.” These behaviours were often described as occurring in the context of heightened stress or emotional breaking point for their child. Unlike the sometimes humorous or wry expressions that accompanied mothers’ reports of their children’s normative resistance, mothers displayed distressed emotions including crying, and expressions of shock or fear.

#### Destroying property

Mothers reported examples of their children destroying property including “kicked a hole in the wall” (Family 22, 10-year-old son), “breaking a window” (Family 1, 13-year-old son) and “cutting up a picture of us” (Family 10, 12-year-old daughter). Many of the incidents reported were extreme and mothers reported being shocked and disbelieving that their child was responsible for the action. For example, one mother reported,

I was scared, he is sitting on the couch with a lighter. A lighter! I am like oh god how did he get a lighter. So, I ask him, S what you got there. And when he looked at me, it was like, ‘Oh god we are in trouble!’. Like I remember thinking to myself, ‘holy shit this isn’t my kid!’. Honestly it was just so unbelievable. He didn’t seem himself at all… he lights the picture frame on fire. (Family 17, 13-year-old son)

#### Physical aggression

Most mothers reported that their child used physical aggression in interactions with parents, peers, and family members. For example, some mothers reported that their children were aggressive at school. One mother described her son getting into a physical altercation with a peer in the playground, “So it turns out he beat this kid to a pulp, it was really bad… because the kid was hospitalized” (Family 17, 13-year-old son) and another mother reported “He received an in-school suspension for cutting a little girl’s hair off in his class.” (Family 3, 12-year-old son).

Mothers also reported aggression towards themselves or other family members. For example, one mother reported that her son attacked her, “He has, he’s been very aggressive at times. So that’s the one when he has come at me… but he would just run at me and come at me” (Family 1, 13-year-old son).

#### Verbal coercion

Verbal coercion was the most common form of extreme aggression reported by mothers. Mothers reported that their child displayed anger at them by yelling, shouting, or screaming. For example, one mother reported how when her daughter became upset she would start “screaming at the top of her lungs… we had to stop the car and like everyone was staring at us in the parking lot because this child sounded like she was being, her toenails or fingernails were being ripped out one by one” (Family 7, 8-year-old daughter). Another mother reported, “But a serious temper tantrum where he will yell at me, and get really angry and storm off to his room or yell hurtful things” (Family 2, 8-year-old son). These instances of verbal coercion appeared to occur when something did not go the child’s way, or if the child was attempting to express some sort of distress.

#### Self-harm

A less frequent form of behaviour reported by mothers classed as extreme aggression was self-harm. Mothers interpreted these as acts of distress, when children were struggling to communicate their emotions. For example, one mother reported,

the worst part is after all this he got really quiet, sort of curled up, and very slowly started talking about not wanting to be in this world (crying)…who trains a parent for this? We have never been suicidal. (Family 22, 10-year-old son)

Mothers reported feelings of intense emotion and fear when their child engaged in these behaviours. One mother described how her daughter had overdosed on prescription medication. She explained “that was a time that I felt I had completely lost her. The choice she made was one that I, to this day, I just can’t figure it out. There’s nothing I can say or do” (Family 10, 12-year-old daughter).

### Parental explanations for extreme aggression

Mother’s explained their child’s extreme aggression in two different ways—*mental health attributions* and *losing control*. These attributions contrasted the normalizing attributions that they used to explain normative resistance. Instead of considering the behaviour as a manifestation of children’s growing autonomy, mothers perceived instances of destructiveness, and verbal and physical aggression to be a symptom of a mental disorder or occurring in a moment when children lost touch with their intentional agency.

#### Mental health

Many mothers explained their child’s extreme aggression by attributing it to their child’s mental health diagnosis, such as conduct disorder, anxiety or depression. For example, one mother explained her daughter’s aggression in relation to her conduct disorder. She explained how she should have predicted her child’s aggression due to previous experiences with her conduct disorder “with a conduct disorder. You can often see it coming because she is so aggressive, or she acts out in a way that is usually noticed” (Family 6, 11-year-old daughter).

Another mother explained that her child’s extreme aggression towards herself and his brother as an unintentional consequence of her child’s mental health. “I don’t think he does it to be mean to me, or like he is doing it because he actually hates his brother. I think he has another stuff going on like his mental health stuff…this isn’t going anywhere good” (Family 25, 11-year-old son). One mother used the metaphor of an uncontrolled canoe to explain parenting a child with anxiety. She said, “having a kid with anxiety or behaviors is like jumping into a canoe with no paddles, no life jacket, and going on the river. Because you are basically stuck in that canoe, like you can’t get out and you are the only two in there” (Family 10, 12-year-old daughter).

Mental health explanations appeared to enable mothers to avoid blaming the child and consider factors outside of the child’s intentional control as influences on children’s aggressive behaviours. One mother reported, “But I think it’s the most common part of conduct disorders, saying no, being aggressive, it goes hand and hand. So I think that it’s not about D, it’s about what’s going on around her” (Family 10, 12-year-old daughter).

#### Losing control

Many mothers stated that when their children were at a heightened point of their distress, their children’s coercive behaviour could dramatically escalate so as to be out of their voluntary control. This was described as children going from “one to a million” (Family 10, 12-year-old daughter), “it’s like she can’t slow it down or she doesn’t know how to. It’s totally a 0–10 sort of thing” (Family 8, 8-year-old daughter), or “in a trance” (Family 16, 12-year-old daughter). Mothers described the change in their child as a “light switch” (Family 25, 11-year-old son) and a “breaking point” (Family 24, 12-year-old daughter) in which the child changed from expressing extreme aggression to “totally losing themselves” (Family 12, 13-year-old daughter).

Some mothers perceived that if they unable to maintain a personal connection with their child during such episodes, at some point an aggressive spiral was inevitable. For example, mothers reported, “You missed your chance to fix this and now it’s going to war” (Family 25, 11-year-old son), “When he is going, if we don’t get to him in time, it’s like, ‘See ya later’. Seriously though, he is totally not himself and we can’t catch up with him” (Family 22, 10-year-old son), and “Bracing yourself for the storm, you know, it’s like, what’s that saying, battle down the hatches” (Family 16, 12-year-old daughter).

Mothers described that when the point of no return occurred, their children were no longer in touch with themselves, and did not have full control over their actions. Mothers reported that their child was “reaching a certain point that she’s just not there” (Family 24, 12-year-old daughter), or “loses herself” (Family 8, 8-year-old daughter), and “can’t see past herself” (Family 6, 11-year-old daughter). For example, one mother reported,

Seriously it’s the worst…. and I honestly don’t think he even knows that he’s doing it, it’s like S, are you in there, is that you. Because it’s sort of just (sighs) it’s just like not him. He isn’t that kid who is trashing our house, swearing at his brother, calling me a fucker. Like where does he learn that language, my god. But even like I said, the look in his eyes, it’s like, hey S are you in there? (Family 25, 11-year-old son)

### Parental responses to extreme aggression

Mothers reported using a drastically different approach to managing children’s nonconforming behaviours when their children engaged in extreme aggression than when they engaged in normative resistance. Rather than attempting to control the child’s behaviour directly, mothers responded to incidents of extreme aggression by responsively attending to the child’s emotional distress or relationally engaging with the child. Mothers their responses to their child’s extreme aggression in three different ways—*verbally reassuring, physically reassuring* and *making relational contact*.

#### Verbally reassuring

Mothers reported that they attempted to calm and support their children with verbal reassurance when children engaged in extreme aggression. For example, one mother described how she communicated support and security “Just let her know that we are here, we are not going anywhere, we are going to stay here even though you are acting like this but when you calm down we are still going to be her” (Family 7, 8-year-old daughter). Another mother described her child’s response to verbal reassurance, “She hears me and can slow down because I am supporting her to slow down” (Family 6, 11-year-old daughter).

One mother described her efforts to communicate with her son in a way that let him know that although his behaviour was not appropriate, he could still be confident of parental support.

I want S to know that it is definitely not okay to hurt someone or hit them, and that he can make better choices during that time. But I also want him to feel like he can come to me, and talk to me, and you know, just that we are in it together. So it’s like, I don’t want to yell at him because it’s not helpful, but I don’t want him to think that when he hits and we are talking that it means it is okay. (Family 22, 10-year-old son)

#### Physically reassuring

Mothers reported that they attempted to communicate reassurance physically by making a physical connection such as a hug, a touch on the arm, or kiss as a way to soothe her child during incidents of extreme aggression. For example, one mother described, “I’d start to rub her back, her you know pet her hair or whatever. I wouldn’t say anything; I’d just stay there” (Family 24, 12-year-old daughter). Another mother staid, “I guess just like kind of being close to her or touching her or snuggling or whatever, it seems to help” (Family 16, 12-year-old daughter) or one mother reported, “If I can get to her to, like if she isn’t tearing around our house, if I can give her a hug and get her to sit calmly with me. That is usually the best way about it” (Family 20, 10-year-old daughter). These mothers reported that some sort of physical reassurance helped their children to de-escalate.

#### Making relational contact

Mothers also described using their physical presence to maintaining an interpersonal connection when their child was out of control without necessarily making any physical contact. Mothers reported that communicating their presence either by being close to the child or by making eye contact, contributed to the child’s ability to de-escalate. For example, one mother described a situation in which her son had picked up the ladder from his bunk bed and was threatening to hit her with it. She explained that,

That was the moment, I had been really working on it and saying that I have to be firmer and stronger. It was that moment and I stood there, and I did not move. He was holding that thing up and I didn’t move, I stayed there. I knew if I could just get him to look me in the eye, that he wouldn’t do it. That he would remember I was his Mom. That is probably the last time he actually did that. (Family 1, 13-year-old son)

This mother used visual contact to remind the child of their personal relationship and change the outcome of a potentially violent. Other mothers also described using relational contact to change the course of extreme aggression. For example, one mother reported “So I just stay with her or near her, which is like hiding behind a chair in case she throws something, but she can always see me when she’s like that. And eventually she slows down” (Family 6, 11-year-old daughter) and another mother explained, “he like glares at me and pushes me out of the way to storm upstairs…So I just tried to be with him, even though I was totally terrified. I stayed near him, I was like okay if he knows I am nearby… it will make a difference” (Family 17, 13-year-old son).

## Discussion

This study provided insights into the nature of the nonconforming behaviours experienced by mothers who sought clinical services for their children’s hard to manage behaviour. Theory, empirical research, and clinical interventions have revolved around the construct of “noncompliance” and the assumption that such behaviour is the consequence of incompetent parenting or unskillful discipline (Kalb & Loeber, 2003; Patterson, 2005). The analyses of this study were approached with a broader conceptual framing than what has been used previously, sensitized not only by longstanding behavioural conceptions of noncompliance (Patterson, 1982) but also alternative conceptions of child resistance from developmental psychology that emphasize children’s legitimate and strategic expression of agency in the face of intrusions on their autonomy (Kuczynski & De Mol, 2015). The results indicated that all mothers in this study identified both normative resistance and extreme aggression as two distinct categories of challenging behaviours in the same children. Mothers’ descriptions of the two phenomena, their causal attributions and their ways of handling the behaviours were qualitatively different.

Mothers described normative resistance as an expected part of children’s developing autonomy. Mothers reports of ignoring, assertive refusal, delaying, and expressions of attitude corresponds to findings of children’s resistance strategies in non-clinical families during early and middle childhood (Kuczynski & Kochanska, 1990; Kuczynski et al. this volume). Mothers attributed normative resistance to typical developmental processes or their children’s assertive personality (Kuczynski, Robson, Burke, & Song, 2015). Lastly, mothers’ responses to normal resistance in this clinical sample were similar to childrearing strategies found in previous research on non-clinical families (Kalb & Loeber, 2003; Kuczynski & Hildebrandt, 1997) including exercising firm control and administering consequences, using proactive strategies to prevent resistance, and supporting children’s appropriate expression of assertion and autonomy.

The same mothers also identified a problematic form of nonconformity, extreme aggression, and their descriptions of physical and verbal coercion and property destruction property, which were consistent with the clinical literature (Matthys & Lochman, 2017; Patterson, 2005). In addition, mothers’ descriptions of dysregulated emotion and escalating, out of control lashing out behaviour is consistent with emerging research on autonomic reactivity, emotional liability and coercive behaviour associated with noncompliance in clinical populations (Beuchaine & Zalewski, 2016).

An important finding that was inconsistent with the expectations from a clinical behavioural perspective was the way that mothers’ handled instances of extreme aggression.

Very few mothers firmly confronted children when they exhibited extreme aggression and no mothers reported using time out procedures as prescribed by standard behavioural approaches to this behaviour (McMahon & Forehand, 2003). Instead, mothers avoided control and used relational strategies such as physical and verbal reassurance and maintaining a physical presence for their children. Indeed, mothers appeared to behave in a way consistent with attachment perspectives (Bowlby, 1969, 2005) by approaching children as agents, addressing children’s needs for security by assuring them of their support and providing responsive care to alleviate their children’s emotional dis-regulation. Rather than treating these challenging child behaviours as intentional acts of aggression (Patterson et al., 1992), mothers attributed their behaviours to a lack of control by their child and acted responsively with empathic care. Such recourse to relational contact is inconsistent with manualized prescriptions of demands for immediate compliance or aversive consequences such as time out in response to defiance or aggression (Forehand, Lafko, Parent, & Burt, 2014).

The distinction between two different child behaviours, attributions, emotions, and strategies for each challenging behaviour existing in the same families has theoretical implications. The results of this study suggested that both types of noncompliance exist in the same families and thus both literatures may be at least partially relevant. In these results, it was evident that children’s resistance was at times expected, tolerated or even welcomed by parents, as expressions of their children’s developing agency and assertiveness. Thus, a direction for future research is to explore how insights from developmental psychology and clinical psychology could be integrated in the aetiology and treatment of children exhibiting challenging behaviours.

In summary, this study suggests that mothers had a complex view of their children’s agency as it relates to health and well-being. Most models of agency including social relational theory emphasize the constructive function of child agency in self-regulation, and self-determination, and as well as the children’s contributions of to their own development and the development of parents. From this standpoint, how is the agency of children exhibiting dysregulated emotion and extreme aggression to be understood? In mothers’ perception, during moments of extreme aggression their children lost the ability to act in an intentional manner. This perception was conveyed by expressions such as “lost control” and “not there” and not himself.” Although intentionality is not a prerequisite for agency, such disordered expression of agency, have not seriously been considered by social relational theory or by classic behavioural accounts of noncompliance in which children are often described as acting coercively to achieve their goals (Patterson, 1982).

One way of understanding extreme aggression is to consider social relational theory’s treatment of the interplay of agency and power. According to social relational theory children are understood to be agents whose effectiveness as agents are backed up by individual, social, and cultural resources (Kuczynski & De Mol, 2015). Thus, children may still be considered as agents when engaging in extreme aggression, but because of their stress or dysregulated emotional state, they were unable to express their agency with full access to executive capacities which regulate intentionality and planning. Particularly interesting from this interpretation were accounts of mothers who in the midst of children’s rage attempted to maintain or establish relational contact with their children. A speculation is that mothers may have attempted to reconnect children to their mindfulness and self-control by reassuring them of their continued access to relational resources stemming from the parent–child relationship. Mothers perceptions of children’s agency and their differential treatment of behaviours they interpret as intentional, unintentional or out of control is a concept that requires further exploration.

The primary limitation for this study was the general nature of inclusion criteria. The diversity and variety in experiences and support systems accessed by the participants may have influenced the results. Also, this research should be conducted in other cultural contexts. These results may be culturally specific to a Canadian context. Another consideration for this study is the composition of the sample. It is important to acknowledge the different family compositions of this sample and consider the complexity of these compositions. Many of these families were middle class, had attained university education and had strong support systems and thus appear to have greater resources than the multi-stressed families that have been described in the clinical literature (Patterson et al. (1992). Further examination of family compositions and characteristics could provide an exciting opportunity to add complexity to traditional, deficit based depictions of families who access services for children’s challenging behaviours.

The results of this constructivist grounded theory study provides several directions for future research and theorizing. First, it was evident that both behavioural (Patterson, 1982) and developmental theories (Kuczynski & De Mol, 2015) were useful for understanding various aspects of mothers’ experiences of parenting and child agency. Further exploration is required to examine in more detail the ways that these two theoretical perspectives could be amalgamated to gain a more holistic understanding of parent-child relationships and child agency. Researchers could explore more specifically which parts of each theory are complementary, and the implications of combining or utilizing multiple theories to inform a practical approach. Lastly, this research could be helpful for practitioners, mental health workers, and individuals working in parent support systems. First, to further explore the concept that noncompliance is not universally maladaptive. This finding encourages consideration of two qualitatively different forms of resistance, which led to two different kinds of responses for mothers. Researchers and practitioners could continue to examine the ways that parents are experiencing this phenomenon and what it might look like in a more diverse sample.
